# Src controls castration recurrence of CWR22 prostate cancer xenografts

**DOI:** 10.1002/cam4.144

**Published:** 2013-10-11

**Authors:** Bing Su, Bryan Gillard, Lingqiu Gao, Kevin H Eng, Irwin H Gelman

**Affiliations:** 1 Biomedical Research Institute, Shenzhen PKU-HKUST Medical CenterShenzhen, Guangzhou, China; 2 Department of Pharmacology and Therapeutics, Roswell Park Cancer InstituteBuffalo, New York; 3 Department of Cancer Genetics, Roswell Park Cancer InstituteBuffalo, New York; 4 Department of Biostatistics and Bioinformatics, Roswell Park Cancer InstituteBuffalo, New York

**Keywords:** Castration-recurrent prostate cancer, CWR22, dasatinib, KXO1, shRNA, Src, xenografts

## Abstract

Recurrence of prostate cancer (CaP) after androgen-deprivation therapy continues to have the greatest impact on patient survival. Castration-recurrent (CR)-CaP is likely driven by the activation of androgen receptor (AR) through multiple mechanisms including induction of AR coregulators, AR mutants or splice variants, and AR posttranslational modification such as phosphorylation by Src-family and Ack1 tyrosine kinases. Here, we address whether Src is required for the CR growth of human CWR22 CaP xenografts. The shRNA-mediated Src knockdown or treatment with the Src inhibitors, dasatinib or KXO1, reduced CaP recurrence over controls and increased time-to-recurrence following castration. Moreover, CR-CaP [Src-shRNA] tumors that recurred had similar Src protein and activation levels as those of parental cells, strengthening the notion that Src activity is required for progression to CR-CaP. In contrast, the ability of dasatinib or KXO1 to inhibit Src kinase activity in vitro did not correlate with their ability to inhibit serum-driven in vitro proliferation of CR and androgen-dependent stable cell lines derived from CWR22 tumors (CWR22Rv1 and CWR22PC, respectively), suggesting that the in vitro proliferation of these CaP lines is Src independent. Taken together, these findings strongly suggest that Src is a potent and specific therapeutic target for CR-CaP progression.

## Introduction

Prostate cancer (CaP), the second leading cause of cancer deaths in U.S. men (http://seer.cancer.gov/statfacts/html/prost.html), progresses from localized to invasive disease associated with metastasis to local lymph nodes and bones. Unlike the high cure rate of early, localized disease, the so-called lethal clinical phenotype of CaP relates to recurrence following androgen-ablation therapy, producing castration-recurrent (CR) CaP that responds poorly to standard chemotherapy and radiation [1]. Multiple studies indicate that the vast majority of CR-CaP cases present with increased protein and activation levels of WT-AR (wild-type androgen receptor) [2]. This is thought to facilitate AR-driven tumor progression in response to the postcastration expression of low tissue androgen levels [3]. AR mutations and splice variants have been described that can facilitate AR activity in the CR setting [4, 5], yet other mechanisms leading to the activation of AR in CR-CaP have been described, including AR stabilization [6], induction of AR coregulators and posttranslational modification [7].

Several Src-family tyrosine kinases (SFK) are overexpressed and activated in a wide variety of human cancers, including metastatic- or CR-CaP [8–10]. Progression to androgen independence in human (prostate cancer cells) LNCaP is associated with an increased interaction of activated Src with AR [11–13]. Additionally, increased levels of activated receptor tyrosine kinases in CaP progression, known to activate downstream SFK, is associated with increased androgen-independent AR activation [14]. Src activity is also required for the increased expression and secretion of neuropeptides, such as bombesin or interleukin (IL)-8, by prostate neuroendocrine cells to induce androgen-independent growth in LNCaP cells [15, 16]. Guo et al. [17] showed that Src could directly phosphorylate Src on Y534 and that this modification facilitated the CR growth of CWR22R1, a cell line derived from recurrent growths of human androgen-dependent (AD) CWR22 xenografts in castrates [18], or for androgen-independent growth induced by neuroendocrine-derived parathyroid hormone-related protein [19]. The Ack1 nonreceptor tyrosine kinase can also activate AR by the direct phosphorylation on Y267 [20]. Newer studies have corroborated the involvement of activated Src in progression to androgen independence [21–23] or increased invasiveness [24]. These data as well as preclinical studies demonstrating critical roles for SFK in CaP metastasis [25–30] have spawned clinical trials using SFK-specific or pan-tyrosine kinase inhibitors (reviewed in [1, 31]).

The CWR22 xenograft model has been used extensively to study AR-driven postcastration recurrence [32]; however, the role of Src in CR progression has not been studied. Using Src-specific shRNA, the pan-tyrosine kinase inhibitor, dasatinib, and the SFK/tubulin inhibitor, KXO1, we show that Src antagonism decreased spontaneous CR-CaP formation and increased time to recurrence. Moreover, CR-CaP lesions recurring after Src shRNA-mediated knockdown exhibited control Src protein and activity levels, suggesting that sustained Src activity is required for CR growth in this model. These data suggest a role for SFK targeting in some CR-CaP.

## Material and Methods

### Antibodies and reagents

The following primary antibodies (Ab) were used: rabbit polyclonals specific for GAPDH (Santa Cruz Biotechnology, Santa Cruz, CA), Lyn, Paxillin, Paxillin^poY118^, Src^poY416^ (Cell Signaling Technology, Beverly, MA), and mouse monoclonal specific for Src (Src-1; Oncogene Sciences). Secondary antibodies included horseradish peroxidase-labeled anti-rabbit or anti-mouse Ig (EMD-Millipore, Billerica, MA). All reagents were from Sigma (St. Louis, MO) unless stated otherwise.

### Cell culture and virus production

HEK293T cells (ATCC CRL-11268) were maintained in Dulbecco's modified Eagle's media (DMEM) supplemented with 10% heat-inactivated bovine serum (BS). Lentiviruses were produced by transient transfection of HEK293T cells with shRNA-encoding lentivirus vector DNA plus DNAs encoding VSV (vesicular stomatitis virus)-G envelope (pMD2G) and HIV-based Gag, Pol and regulatory proteins (pCMV-R8.74), gifts of Didier Trono (École Polytechnique Fédérale de Lausanne, Lausanne, Switzerland).

### Proliferation assay

CWR22Rv1 and CWR22PC plated in triplicate in RPMI-1640 plus 10% fetal calf serum (fCS) at a density of 2000/well in 96-well plates were treated for 72 h with increasing concentrations of KXO1 or dasatinib (from 1 nmol/L to 100 μmol/L), or vehicle (dimethyl sulfoxide, DMSO), then stained with 3-(4,5-dimethylthiazol-2-yl)-2,5-diphenyltetrazolium bromide using the Vybrant® MTT Cell Proliferation Assay Kit (Promega, Madison, WI).

### Growth of CWR22 xenografts

One week before tumor cell inoculation, sustained-release testosterone (T) pellets (12.5 mg/kg in silastic tubing) were placed s.c. between the shoulder blades in castrated athymic nude male mice. Each mouse was injected s.c. with 10^6^ cells in 100 μL of Matrigel (5 mg/mL; Bedford, MA) in phosphate buffered saline (PBS). Tumor volumes and mouse weights were measured weekly, and the tumor volumes were calculated using the formula, L × L × W × 0.5234. When the tumors reached ∼250 mm^3^, the T pellets were removed and treatment (orally once daily, sid po) started the next day for 28 days with dasatinib (15 mg/kg), KXO1 (10 mg/kg) or vehicle (80 mmol/L sodium citrate/citric acid buffer, pH 3.0). Tumor volumes were measured every 2 weeks for recurrence. To assess the ability of dasatinib or KXO1 to inhibit Src kinase activity in tumors, CWR22-tumored (250–400 mm^3^), T-pelleted male nude mice were treated for 2 weeks with sid po doses of dasatinib, KXO1 or vehicle, as above. Mice were sacrificed 3 h after the final dosing, and tumor lysates were prepared immediately.

### Transduction of CWR22

AD CWR22 primary tumors grown in T-pelleted nude male mice were harvested at volumes of 250–400 mm^3^, and tumor tissues were digested with collagenase type I (Invitrogen-Life Technologies, Grand Island, NY). After washing the cells twice with sterile PBS, 10^6^ cells/6-cm dish were plated and then infected after 4 h of attachment with 1 mL of DMEM containing lentiviruses (scrambled-shRNA or Src-shRNA [17]; gifts of Zhiyong Guo, University of Maryland School of Medicine) at a multiplicity of 2.5 (based on expression of viral Green Fluorescent Protein [GFP]). Infection was facilitated by centrifugation at 2500 × g for 30 min in a swinging bucket rotor.

### Immunoblotting

Immunoblotting (IB): The tumor tissues were homogenized and lysed in RIPA (radioimmunoprecipitation assay) buffer (10 mmol/L Tris, pH 7.4, 150 mmol/L NaCl, 5 mmol/L EDTA (ethylenediaminetetraacetic acid), 8% glycerol, 1% Triton X-100, 0.1% SDS, 0.5% sodium deoxycholate) supplemented with 10 mmol/L Na_3_VO_4_, 1 mmol/L NaF, and Complete Protease Inhibitor Cocktail (Roche Diagnostics, Mannheim, Germany). A quantity of 40 μg total proteins per sample was separated by SDS-PAGE (sodiumdodecyl sulfate polyacrylamide gel electrophoresis), blotted onto PVDF (polyvinylidene difluoride) membranes, which were blocked for 30 min with 5% BS albumin in 1× TBS/T (0.1% Tween-20 in Tris-buffered saline) probed with primary and secondary Abs (including three TBS/T washes after each Ab step), and then imaged after incubation with Lumi-Light chemiluminescence reagent (Roche) as described previously [33]. Digital imaging and signal quantification were performed using a Chemi-Genius^2^ Bio-Imager (Syngene, Frederick, MD) and GeneTools software.

### Statistical analyses

Differences in time to tumor recurrence between treatment and vehicle control were compared using Kaplan–Meier curves and the log-rank test. Mean time to recurrence is computed from the area under the curve truncated at 220 days (restricted mean life). The *P*-value for the comparison of Src shRNA is based on a chi-square test.

## Results and Discussion

### Src inhibitors decrease CWR22 tumor recurrence

**Figure 1:**
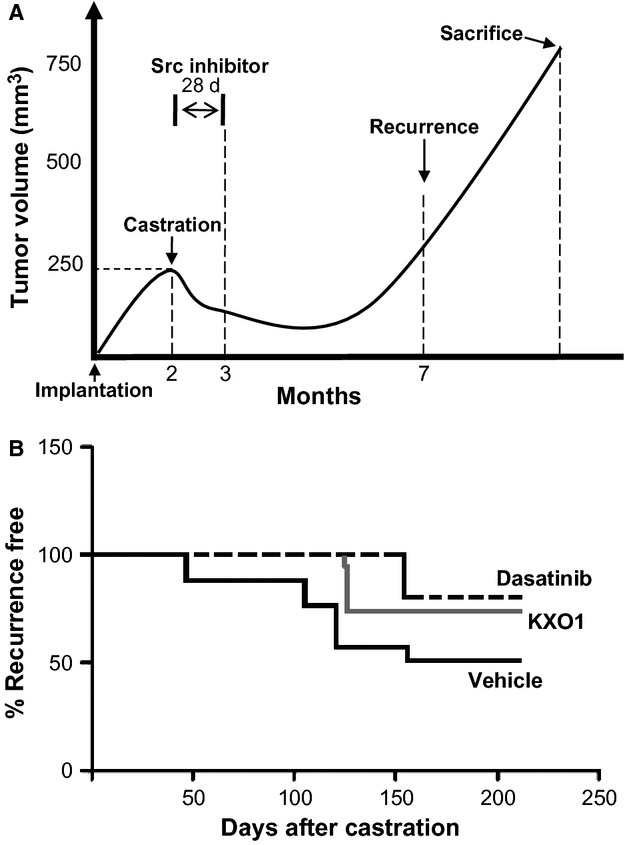
Src inhibitors decrease the frequency and rate of CWR22 recurrence. (A) Schematic view of the treatment protocol in regard to CWR22 androgen-dependent (AD) and castration-recurrent (CR) growth. CWR22 xenograft cells are implanted in castrated, T-pelleted male nude mice, and after the tumor reaches a volume of roughly 250 mm^3^, the pellet is removed (“Castration”). Most tumors either regress somewhat or stop growing over the next month, a period in which the mice were either treated with dasatinib, KXO1 or vehicle. Non-recurrence was defined as no net tumor growth in a 7-month period, based on the regressed tumor size following castration. Recurrence was defined as progressive tumor growth within 7 months of castration to sizes equal to or greater than those at precastration levels. With no treatment or vehicle, 40–50% of the tumors started to recur between 3–5 postcastration. (B) Frequency and rate of CWR22 recurrence postcastration (T-pellet removal from castrated mice) recurrence in vehicle, dasatinib or KXO1-treated groups (*n* = 20).

The well established human CWR22 xenograft model [18] can recapitulate initial AD CaP growth in vivo followed by castration-induced regression and the recurrence in 40–50% of hosts of CR-CaP that express and are driven by AR [32] (Fig. 1A). Recurrence is defined by the growth of the primary-site (s.c.) tumor after a postcastration regression to volumes comparable to (and eventually, greater than) precastration levels (Fig. 1A). Nonrecurrence is defined as no net growth over regressed tumor volumes during the 7-month postcastration period. It should be noted that CR-CaP lesions as well as a cell line derived from these lesions, CWR22Rv1, express mutated version of AR (H874Y) from the parental CWR22 [34], as well as a truncated AR which exhibits constitutive nuclear localization and DNA-binding activity [35]. Importantly, these forms are still likely regulated by Src as CWR22Rv1 growth in castrated nude mice requires Src [17].

In order to determine whether Src is required for the spontaneous generation of CR-CaP in the CWR22 model, CWR22 xenografts were grown to ∼250 mm^3^ in T-pelleted, castrated male nude mice, then treated for 28 days (starting 1 day before T-pellet removal) with doses of dasatinib or KXO1 (vs. vehicle) (Fig. 1A) previously shown to inhibit Src-driven tumor growth in vivo [10, 36]. Compared to controls, dasatinib and KXO1 had no effect on postcastration tumor regression (Table 1), suggesting that this process is SFK independent. In contrast, KXO1 and dasatinib decreased overall CR-CaP formation by 60% or 50%, respectively (Table 1), and although these decreases may not be statistically significant, the ability of KXO1 and dasatinib to delay the time-to-recurrence of CR-CaP (Fig. 1B) by 1 or 2 months, respectively, showed strong statistical power (Table 1).

**Table 1:** Effect of KXO1^1^ and dasatinib^2^ on tumor occurrence.

Group	Regression^3^	Recurrence	Mean time to recurrence (SE)^4^	Log-rank test vs. vehicle^5^
Vehicle	7.8days (1.6)	10/20 (50%)	164.3 (13.4)	
KXO1	8.2days (1.6)	4/20 (20%)	206.0 (6.3)	*P*=0.0494
Dasatinib	7.2days (1.2)	5/20 (25%)	200.0 (7.8)	*P*=0.0225

1 Ten milligram per kilogram po sid for 28days after castration.

2 Fifteen milligram per kilogram pos id for 28days after castration.

3 Days postcastration for maximal regression or growth arrest (SE).

4 *P*-values for the recurrence percentages are vehicle versus KXO1 (*P*=0.097) and vehicle versus dasatinib (*P*=0.191) with standard error (SE) based on the standard chi-square tests.

5 The Log-rank tests compare the difference between individual survival curves. The overall test (any difference between the three) is *P*=0.0261.

**Figure 2:**
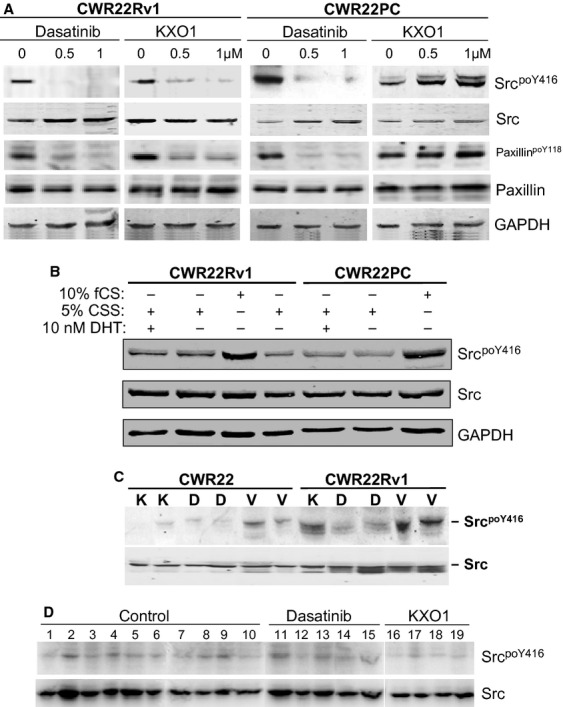
Effect of dasatinib or KXO1 on Src activation levels in vitro and in vivo. (A) Lysates of CWR22Rv1 or CWR22PC cells grown in media with 10% fetal calf serum (fCS) and treated for 24 h with vehicle (“0”), dasatinib or KXO1 (0.5 or 1 μmol/L) were immunoblotted for total Src, Src^poY416^, Paxillin, Paxillin^poY188^ or GAPDH (as a protein loading control). NS, nonspecific band. (B) Cells grown for 72 h in 5% charcoal-stripped serum (CSS) were treated with 10 nmol/L dihydrotestosterone (DHT) (24 h) or 10% fCS (1 h), and lysates were then immunoblotted as in panel (A). (C) T-pelleted nude male mice carrying CWR22 tumors or castrated nude mice carrying CWR22Rv1 tumors were treated for 14 days with vehicle (V), dasatinib (D) or KXO1 (K), and then tumors were excised 3 h after the last treatment. Tumor lysates were analyzed for total- or poY416-Src as in panel (A). (D) Castration-recurrent prostate cancer (CR-CaP) tumors arising from mice treated for 28 days right after castration with dasatinib (tumors 11–15), KXO1 (tumors 16–19) or vehicle (tumors 1–10), as described in Table 1, were analyzed for Src and Src^poY416^ by immunoblot as above.

The ability of dasatinib and KXO1 to inhibit Src activation in cultured CWR22Rv1 cells was demonstrated by comparing total Src protein to autophosphorylation levels, marked by Src^poY416^ (Fig. 2A, upper panel), previously established as a surrogate marker for Src kinase activity levels in cells [37]. Src activation levels in CWR22Rv1 cells were inhibited by 1 μmol/L dasatinib or KXO1; however, whereas dasatinib suppressed Src activation levels in CWR22PC, an AD cell line derived from primary CWR22 xenografts [38], KXO1 induced Src activation levels in these cells, both at the level of Src autophosphorylation and transphosphorylation of the substrate, paxillin, on Y118 (Fig. 2A). This may reflect different mechanisms of action by the two compounds: dasatinib is an ATP-competitive kinase inhibitor [39] whereas KXO1 is a non-ATP-competitive peptide binding-site inhibitor [40, 41]. How this is manifest remains unclear because Src activation is equally inducible by serum in both CWR22Rv1 and CWR22PC cells, and equally unaffected by androgen alone (Fig. 2B). Importantly, though, (“AD”: CWR22) and CR (CWR22Rv1) tumors exhibited inhibition of Src activity 3 h after the last dose in a 14-day treatment regimen with dasatinib or KXO1 (Fig. 2C), indicating that Src was targetable by these drugs in the in vivo setting. Additionally, unlike dasatinib, KXO1 may uniquely be able to bind Src complexes and/or conformations that occur only in androgen-sensitive cells in the in vitro setting.

Given the discordance between the ability of dasatinib or KXO1 to inhibit Src activity in vitro versus in vivo, we tested the ability of these drugs to inhibit in vitro proliferation of the AD versus CR CaP lines. Even though dasatinib inhibited Src autophosphorylation level >90% at 1 μmol/L (Fig. 2A), it was poor at inhibiting proliferation in vitro based on growth inhibitory (GI_50_) values of 26 μmol/L or 56 μmol/L in CWR22Rv1 or CWR22PC cells, respectively (Table 2). In contrast, even though KXO1 inhibited Src activity in the CWR22Rv1 cells only (Fig. 2A), it was more potent than dasatinib at inhibiting in vitro proliferation, with GI_50_ values of 232 nmol/L in CWR22Rv1 or 21 nmol/L in CWR22PC cells. Taken together with the data above, this suggests that CR growth in vivo is Src dependent whereas the serum-induced in vitro proliferation of CWR22Rv1 or CWR22PC is either Src independent or the broad specificity of dasatinib negates inhibitory pathways required downstream of Src for proliferation in vitro. It is also likely that the increased ability of KXO1 to inhibit the in vitro proliferation of CWR22Rv1 or CWR22PC might be due to additional non-Src targeting functions, such as pretubulin inhibition, as has been described recently (http://www.kinexpharma.com/drug-pipeline/kx01).

**Table 2:** Growth inhibitory concentrations (GI_50_^1^) of KXO1 and dasatinib.

	CWR22Rv1	CWR22PC
KXO1 (nmol/L)	232	21
Dasatinib (nmol/L)	26,464	56,203

1 GI_50_: drug concentration yielding 50% proliferation inhibition versus vehicle alone.

If CR growth of the CWR22 tumors is Src dependent, then the CR tumors that arise spontaneous or after dasatinib/KXO1 treatment should continue to express activated Src. Thus, relative levels of activated Src were determined in the CR-CaP tumors that arose months after treatment with vehicle, dasatinib or KXO1. All CR-CaP lesions recurring after dasatinib or KXO1 treatment exhibited similar relative Src^poY416^ levels to vehicle-treated CR-CaP tumors (Fig. 2D). This contrasts with data in Figure 2C showing that acute treatment with dasatinib or KXO1 could inhibit relative Src^poY416^ levels. Given that expression of activated Src is sufficient to induce CR-CaP in a tissue recombination model of CaP [24], our results suggest that continued Src activation promotes CR-CaP in the CWR22 model. It remains unclear whether longer drug treatment might be sufficient to suppress CR-CaP generation more completely; however, the finding of sustained Src activation levels in CR-CaP lesions from dasatinib- and KXO1-treated mice strongly suggests that drug resistance in this context is not due to non-Src compensatory mechanisms.

### siRNA-mediated Src knockdown suppresses recurrence of the CWR22 tumor

**Figure 3:**
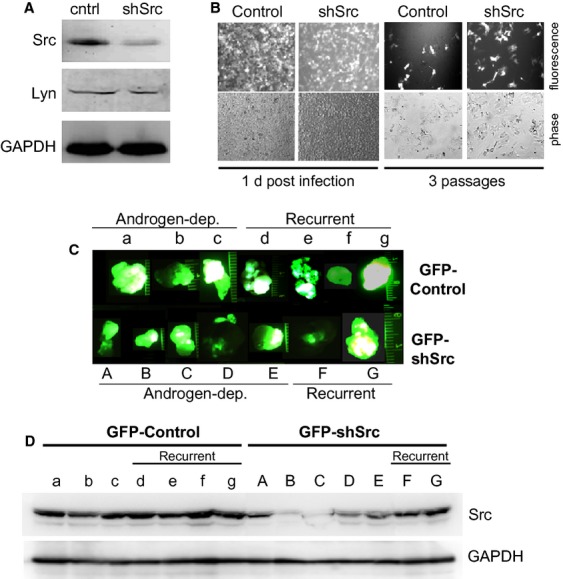
Sustained RNAi-mediated Src knockdown in recurrent CWR22 tumors. (A) Lysates of CWR22Rv1 cells stably infected with lentiviruses expressing Src- or control-shRNA were immunoblotted for Src, Lyn or GAPDH. (B) GFP fluorescence (upper panel) and phase contrast (lower panel) micrographs of androgen-dependent CWR22 tumor cells infected ex vivo with lentivirus expressing GFP as well as control- or Src-shRNA, either 1 day postinfection or after three passages. (C) Sustained GFP fluorescence in androgen-dependent and recurrent tumors formed after reinjection of CWR22 cells transduced with control- or Src-shRNA GFP-expressing lentiviruses (a–c for control-shRNA; A–E for shSrc), and in recurrent tumors (d–g for control-shRNA; F and G for shSrc). (D) Lysates of androgen-dependent (a–c for control-shRNA; A–E for shSrc) or recurrent CWR22 tumors (d–g for control-shRNA; F and G for shSrc) were analyzed by immunoblotting for Src versus GAPDH protein levels.

Although dasatinib was originally described as a Src/Abl-specific inhibitor [39], there is appreciation that it functions as a pan-tyrosine kinase inhibitor [42]. Additionally, KXO1 (also called KX2-391) targets several SFK members as well as Abl, and newer data indicate that it also targets tubulin polymerization (D. Hangauer, Kinex Pharmaceuticals LLC, pers. comm.). Thus, to more specifically address the role of Src in the generation of CR-CaP, we sought to knock down Src expression using lentivirus transduction of Src-shRNA. We first showed that compared to control vector-infected cells, CWR22Rv1 transduced with Src shRNA (“shSrc”) showed significant knockdown of Src protein levels (Fig. 3A) compared to another SFK family member, Lyn. Primary CWR22 tumors grown to ∼250 mm^3^ in T-pelleted male nude mice were excised, converted into single-cell suspensions by collagenase treatment, and after washing, plated and rapidly infected with high-titer lentivirus encoding control- or Src-shRNA at efficiencies close to 100% infection, based on expression of the surrogate lentivirus-encoded GFP marker (Fig. 3B, left panel). Indeed, GFP expression was sustained in vitro even as the cells senesced in culture (after three passages; Fig. 3B, right panel) and after AD tumor growth following reinoculation into T-pelleted male nude mice (Fig. 3C). All recurrent tumors exhibited strong GFP expression (Fig. 3C; tumors d–g in the controls, and F and G in the shSrc group), indicating there was sustained proviral expression 6 months or more after reinjection of the infected CWR22 cells. Paralleling the dasatinib/KXO1 studies above, Src knockdown decreased the spontaneous generation of CR-CaP lesions by 50% (Table 3). Again, although this decrease was not statistically significant (*P* = 0.3 by *χ*^2^ test), there was significance for the 1-month increase in time-to-recurrence in the shSrc versus control groups (*P* = 0.0241). Importantly, loss of Src had no effect on the rates of primary tumor growth or postcastration regression (data not shown), in agreement with our data given above that neither dasatinib nor KXO1 affected postcastration regression (Table 1). The levels of Src protein in five primary, AD shSrc-expressing tumors (Fig. 3D, lanes A–E) was uniformly lower than in primary control tumors (lanes a–c), indicating a sustained effect of the Src shRNA in vivo. In contrast, Src protein levels in the two shSrc CR-CaP lesions (lanes F and G) were similar to those in control primary and recurrent lesions. Although these numbers are small, these data strengthen the concept that Src is required for CR-CaP generation in this system.

**Table 3:** Effect of Src shRNA on tumor occurrence.

Group (*n*=10)	Recurrence	*χ*^2^ test vs. vehicle
Control-shRNA	4/10 (40%)	
Src-shRNA	2/10 (20%)	*P*=0.622

This study is the first to demonstrate a role for Src in the spontaneous generation of CR-CaP using a model that starts with an AD human CaP xenograft. The growing acceptance that Src plays a pivotal role in CaP progression to recurrence and even more specifically, to the formation of bone metastases [43], has spawned multiple clinical studies in CR-CaP using Src inhibitors in conjunction with chemotherapies, such as docetaxel [1, 31, 44–47]. Initial Phase II and Phase I/II studies indicate efficacy for dasatinib alone or in combination with docetaxel using prostate-specific protein (PSA) level and boney metastasis monitoring as therapeutic markers [48, 49]. Data are pending from a current multicenter Phase II trial with KXO1 in CR-CaP cases with boney metastases (NCT01074138).
